# Phosphate-Catalyzed Succinimide Formation from an NGR-Containing Cyclic Peptide: A Novel Mechanism for Deammoniation of the Tetrahedral Intermediate

**DOI:** 10.3390/molecules23092217

**Published:** 2018-08-31

**Authors:** Ryota Kirikoshi, Noriyoshi Manabe, Ohgi Takahashi

**Affiliations:** Faculty of Pharmaceutical Sciences, Tohoku Medical and Pharmaceutical University, 4-4-1 Komatsushima, Aoba-ku, Sendai 981-8558, Japan; hanari0703@yahoo.co.jp (R.K.); manabe@tohoku-mpu.ac.jp (N.M.)

**Keywords:** Asn-Gly-Arg (NGR) motif, deamidation, *iso*Asp-Gly-Arg (*iso*DGR) motif, dual tumor targeting, non-enzymatic reaction, succinimide formation, tetrahedral intermediate, phosphate catalysis, computational chemistry, density functional theory

## Abstract

Spontaneous deamidation in the Asn-Gly-Arg (NGR) motif that yields an *iso*Asp-Gly-Arg (*iso*DGR) sequence has recently attracted considerable attention because of the possibility of application to dual tumor targeting. It is well known that Asn deamidation reactions in peptide chains occur via the five-membered ring succinimide intermediate. Recently, we computationally showed by the B3LYP density functional theory method, that inorganic phosphate and the Arg side chain can catalyze the NGR deamidation using a cyclic peptide, c[CH_2_CO–NGRC]–NH_2_. In this previous study, the tetrahedral intermediate of the succinimide formation was assumed to be readily protonated at the nitrogen originating from the Asn side chain by the solvent water before the release of an NH_3_ molecule. In the present study, we found a new mechanism for the decomposition of the tetrahedral intermediate that does not require the protonation by an external proton source. The computational method is the same as in the previous study. In the new mechanism, the release of an NH_3_ molecule occurs after a proton exchange between the peptide and the phosphate and conformational changes. The rate-determining step of the overall reaction course is the previously reported first step, i.e., the cyclization to form the tetrahedral intermediate.

## 1. Introduction

Since Ruoslahti and co-workers discovered the tumor-homing property of the asparagine-glycine-arginine (Asn-Gly-Arg, NGR) motif by in vivo phage display in tumor-bearing mice [1], peptides containing this motif, especially cyclic ones, have been attracting considerable attention for application to tumor diagnosis and therapy [2,3,4,5,6,7,8,9,10,11,12,13,14,15,16,17,18,19,20,21,22,23,24,25,26,27,28,29,30,31,32,33,34,35,36,37]. The NGR motif binds to an aminopeptidase N (APN or CD13) isoform which is uniquely expressed on the endothelium of tumor neovasculature [3,5].

**Scheme 1:**
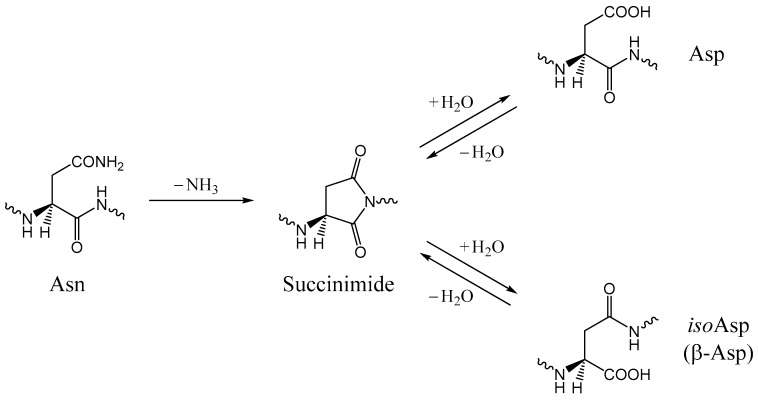
Succinimide-mediated deamidation of an Asn residue giving an Asp or *iso*Asp (β-Asp) residue.

**Scheme 2:**
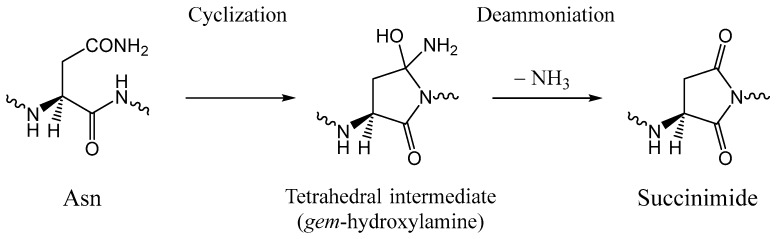
The cyclization-deammoniation two-step mechanism for the succinimide formation from an Asn residue.

It is well known that Asn residues in peptides and proteins undergo spontaneous, non-enzymatic deamidation via the five-membered ring succinimide intermediate (Scheme 1) [38,39,40,41,42,43,44,45,46,47,48]. This intermediate is formed by the nucleophilic attack of the main-chain nitrogen atom of the C-terminal adjacent residue on the Asn side-chain amide carbon (C_γ_) with the release of an ammonia (NH_3_) molecule. This is an intramolecular nucleophilic substitution reaction, and formally proceeds by the cyclization-deammoniation (addition-elimination) two-step mechanism (Scheme 2) [49,50]. In the first step, a so-called tetrahedral intermediate is formed. In the present case, this intermediate is a *gem*-hydroxylamine species having an OH group and an NH_2_ group on the same carbon atom (C_γ_). In the second step, an NH_3_ molecule is released from the tetrahedral intermediate to give the succinimide species. Since the succinimide moiety has two carbonyl groups, its hydrolysis gives either an aspartic acid (Asp, D) residue or an isoaspartic acid (β-aspartic acid) (*iso*Asp, *iso*D) residue. Typically, Asp and *iso*Asp are formed in a ratio of 1:3 [38,39,40,41,42,43,44,48]. Since the succinimide intermediates are racemization-prone, small amounts of d-Asp and d-*iso*Asp residues may also be formed [38,44,48,51,52]. The rates of Asn deamidation are largely dependent on the adjacent amino acid residue on the C-terminal side, and by far the fastest when this residue is Gly [38,40,42,43,44,45,46,48].

The NGR-containing peptides and proteins also undergo spontaneous Asn deamidation to *iso*DGR and DGR [53,54,55,56,57,58,59,60,61,62]. Interestingly, the *iso*DGR-containing peptides, which are the major products from NGR-containing peptides, bind α_v_β_3_ integrin which is also expressed in tumor cells including the endothelium of tumor neovasculature [53,54,55,56,63,64]. Therefore, NGR-containing peptides are expected to be used for dual targeting strategies in specifically targeted delivery of drugs or imaging agents to tumors [10,37,54,56,57,58,59,61,65].

**Figure 1:**
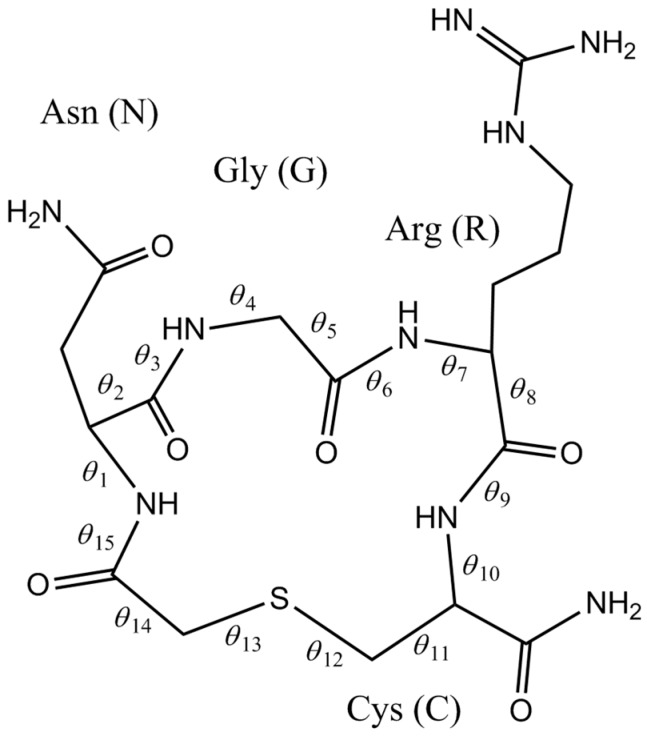
A 15-membered, Asn-Gly-Arg (NGR)-containing cyclic peptide, c[CH_2_CO–NGRC]–NH_2_ (CP15). The guanidino group of the Arg side chain is depicted as a deprotonated form. The definition of the heavy-atom dihedral angles *θ*_1_–*θ*_15_ for the 15-membered ring are also shown.

Recently, Enyedi and co-workers synthesized several NGR-containing cyclic peptides with 15- to 18-membered rings and investigated their deamidation behavior [58]. Among those, we paid attention to a peptide which has a cysteine (Cys, C) thioether linkage in a 15-membered ring, c[CH_2_CO–NGRC]–NH_2_ (Figure 1), because in phosphate-buffered saline (PBS, pH 7.4), this peptide was deamidated most rapidly among those investigated. As suggested by our recent computational studies [52,66], inorganic phosphate is a strong candidate for in vivo succinimide-mediated reactions of Asn and Asp residues. We call this cyclic peptide “CP15”, as in our previous paper [67]. Very recently, CP15 was conjugated with daunomycin, an effective antitumor drug used in the clinic for more than 30 years [59]; the CP15-daunomycin conjugate was shown to undergo rapid deamidation in DMEM (Dulbecco’s modified Eagle medium) cell culture medium containing 10% FBS (fetal bovine serum).

In the previous paper [67], we reported a phosphate-catalyzed mechanism of the succinimide formation from CP15 found by a density functional theory (DFT) computational study. In this previous study, we first postulated that an HPO_4_^2−^ ion interacts with the peptide, in which the Arg guanidino group is protonated, to form a reactant complex, since we were interested in the reaction at physiological pH. However, a proton transfer from the protonated (cationic) guanidino group to HPO_4_^2−^ occured in geometry optimizations, resulted in a reactant complex (RC) between a deprotonated peptide (as shown in Figure 1) and an H_2_PO_4_^−^ ion. This interaction may be consistent with the view by Collins et al. [68] that “specific chemical interactions” should exist between an Arg side chain and phosphate. From this RC, the cyclization to the tetrahedral intermediate occurred, catalyzed by the H_2_PO_4_^−^ ion. In this process, the H_2_PO_4_^−^ ion mediates the double proton transfer that occurs concomitantly with the bond formation to form the five-membered ring. The Arg guanidino group was also shown to play a catalytic role in that it contributes to fix the H_2_PO_4_^−^ ion in the right position. We next assumed that the NH_2_ group on the five-membered ring is easily protonated by the solvent water, and showed that deammoniation occurs from the protonated intermediate with a very low activation barrier. However, the NH_2_ protonation induced a large geometrical change. In the present study, we found a new pathway for the deammoniation process where the initially formed tetrahedral intermediate, which we denote as IC1 (intermediate complex 1) in the present paper, decomposes without an external proton source (and, hence, without changing stoichiometry).

**Figure 2:**
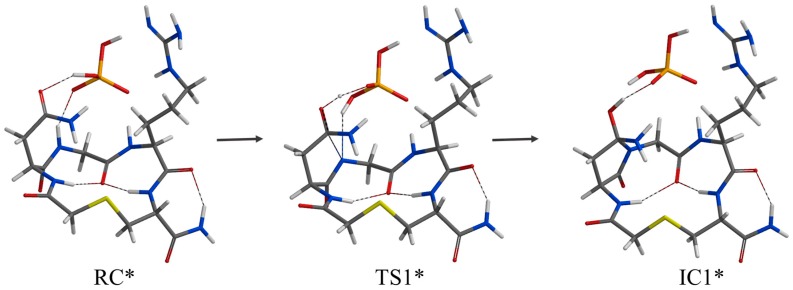
The first step (cyclization to the tetrahedral intermediate) of the succinimide formation from c[CH_2_CO–NGRC]–NH_2_ (CP15) found in the previous study [67]. RC: reactant complex; TS: transition state; IC: intermediate complex. These geometries are labeled by an asterisk (*) to indicate that they can be reproduced from the reported Cartesian coordinates [67] (see text). Grey: carbon; white: hydrogen; blue: nitrogen; red: oxygen; orange: phosphorus; yellow: sulfur.

In Figure 2, we show the geometries in the first step (cyclization): RC (reactant complex), TS1 (transition state 1), and IC1 (intermediate complex 1). In the following, we label these geometries with an asterisk (*) such as RC* to indicate that they can be reproduced from the Cartesian coordinates reported in the previous paper [67]. As shown in Figure 2, RC* is converted to IC1* via TS1*. IC1*, which was previously denoted as TH1 (tetrahedral intermediate 1), is a complex between the *gem*-hydroxylamine tetrahedral intermediate and an H_2_PO_4_^−^ ion.

In the new mechanism revealed here, IC1* decomposes to the product complex (PC), which is formed between the succinimide product, an H_2_PO_4_^−^ ion, and the released NH_3_ molecule, in four steps without a high activation barrier and a large change in the overall geometry. The Arg side chain again plays a catalytic role by fixing the catalytic H_2_PO_4_^−^ ion in the right position to allow the double proton transfers in this four-step decomposition process.

## 2. Results and Discussion

**Figure 3:**
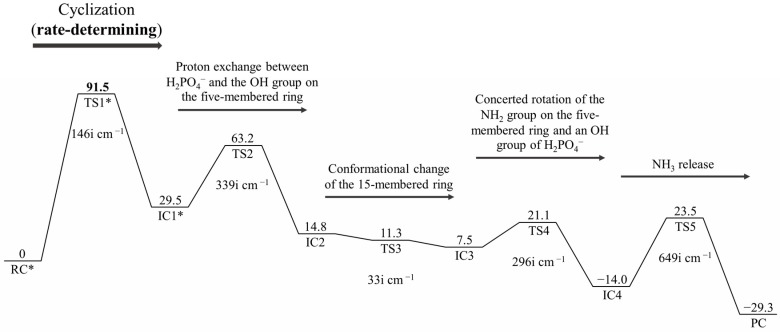
Energy diagram in water at 298.15 K. RC: reactant complex; TS: transition state; IC: intermediate complex; PC: product complex. The geometries labeled by an asterisk (*) (RC*, TS1*, and IC1*) are those previously reported [67]. The relative energies after the thermal and hydration Gibbs energy corrections are shown in kJ mol^−1^ with respect to RC*. Also shown are the imaginary frequencies of the five transition states.

The novel mechanism for the decomposition of the initially formed tetrahedral intermediate (IC1*) comprises four steps. When combined with the previously reported cyclization step [67], the succinimide formation comprises five steps. Figure 3 shows the energy diagram in water for the overall five-step process of the succinimide formation. Geometry optimizations were performed in the gas phase by the density functional theory (DFT) using the B3LYP functional and the 6-31G(d) basis set, followed by vibrational frequency calculations which were used for the zero-point energy (ZPE) calculations and thermal corrections. Moreover, single-point B3LYP calculations using the 6-31+G(d,p) basis set were performed to obtain more reliable electronic energies, and hydration Gibbs energies were estimated by single-point SM8 (solvation model 8) continuum model [69,70] calculations with the 6-31G(d) basis set. The relative energies reported in Figure 3 are those after the thermal and SM8 corrections. These procedures are the same as in the previous study [67] except for the thermal corrections to 298.15 K (see Section 3), by which the activation barrier of the rate-determining first step (see below) increased only by 0.6 kJ mol^−1^. The total energies, ZPEs, and hydration Gibbs energies for the optimized geometries are reported in Table S1. The Cartesian coordinates of the optimized geometries are shown in Tables S2–S9 except for RC*, TS1*, and IC1*, for which they are reported in the previous paper [67].

**Figure 4:**
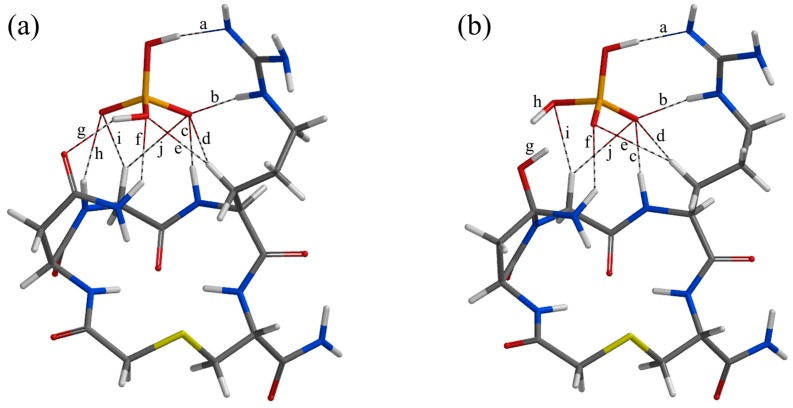
The ten-point interaction (interactions a–j) in RC* (reactant complex) (**a**) and the corresponding interactions (including two O–H covalent bonds (g and h) in IC1* (intermediate complex 1)) (**b**). The corresponding interatomic distances are shown in Table 1. Grey: carbon; white: hydrogen; blue: nitrogen; red: oxygen; orange: phosphorus; yellow: sulfur. These geometries can be reproduced from the Cartesian coordinates reported previously [67].

In RC*, a ten-point interaction is observed between the CP15 molecule (the deprotonated form as shown in Figure 1) and the H_2_PO_4_^−^ ion. These ten interactions (six hydrogen bonds and four CH–O interactions) are shown as interactions a–j in Figure 4a (interactions a, b, c, f, g, and h are hydrogen bonds, and interactions d, e, i, and j are CH–O interactions), and the corresponding interatomic distances are listed in Table 1. In the previous paper [67], we described that there is a nine-point interaction between these two species. However, we now notice an additional weak CH–O interaction (interaction j, 2.911 Å) involving one of the Gly α hydrogens. Interactions a and b are hydrogen bonds between the deprotonated guanidino group and H_2_PO_4_^−^. Interaction c is a hydrogen bond between the main-chain NH group of the Arg residue and H_2_PO_4_^−^. One of the two Arg β hydrogens are involved in interactions d and e, a bifurcated CH–O interaction. It is very important that the C-terminal adjacent residue of Asn is Gly, since one of its α hydrogens that corresponds to the side chain of l-amino acids is involved in a bifurcated CH–O interaction (interactions i and j). If this Gly residue is replaced by another amino acid, there will not be a space to accommodate an H_2_PO_4_^−^ ion because of the steric effect of the side chain. These seven interactions (a, b, c, d, e, i, and j) are maintained throughout the reaction course from RC* to PC, and are thought to be important in the phosphate-catalyzed NGR deamidation mechanism described here. Interactions g and h enable the double proton transfer that occurs in the first step, i.e., the cyclization to the tetrahedral intermediate. Interaction f is a hydrogen bond between the Asn NH_2_ group and H_2_PO_4_^−^, and also contribute to fix the H_2_PO_4_^−^ ion adequately for the first step.

The optimized geometries (other than RC* and TS1*) are shown in Figures 5–13. In Table 2, the dihedral angles *θ*_1_–*θ*_15_ defined for the 15-membered ring as in Figure 1 are shown for all the optimized geometries.

**Figure 5:**
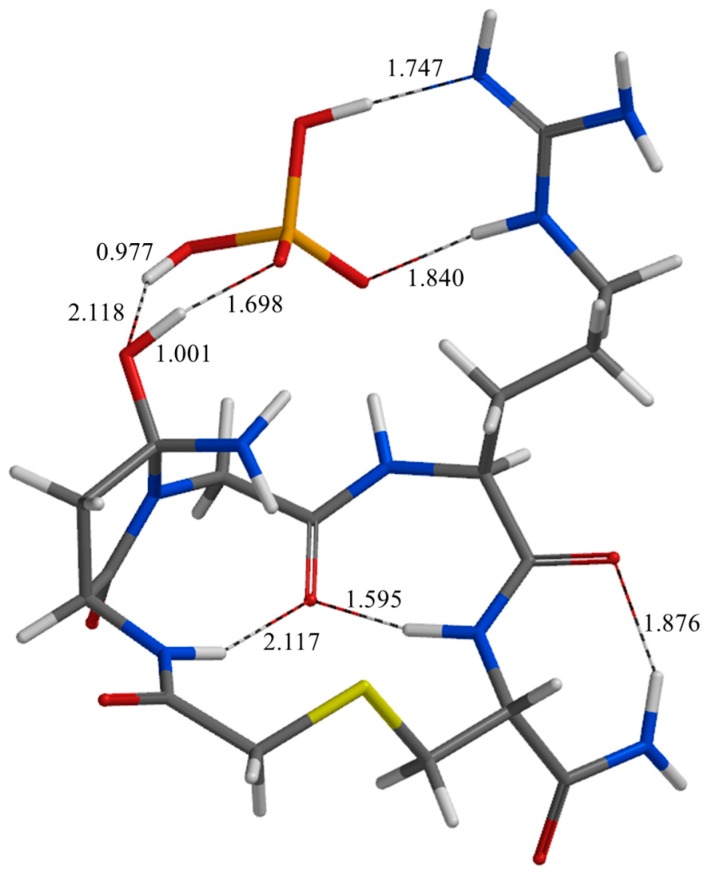
The geometry of IC1* (intermediate complex 1) lying between TS1* (Figure 2) and TS2 (Figure 6). Selected interatomic distances are shown in Å. Grey: carbon; white: hydrogen; blue: nitrogen; red: oxygen; orange: phosphorus; yellow: sulfur. This geometry can be reproduced from the Cartesian coordinates reported previously [67].

**Figure 6:**
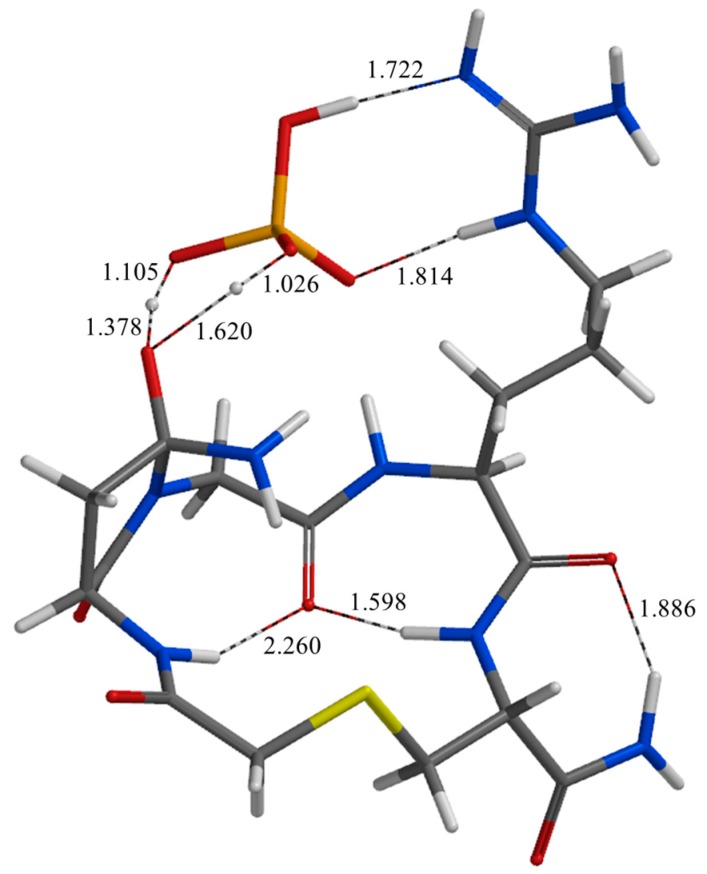
The geometry of TS2 (transition state 2) connecting IC1* (Figure 5) and IC2 (Figure 7). Selected interatomic distances are shown in Å. Grey: carbon; white: hydrogen; blue: nitrogen; red: oxygen; orange: phosphorus; yellow: sulfur.

Figure 5 shows the geometry of IC1* lying between TS1* and TS2 (transition state 2, Figure 6). IC1* is identical with TH1 reported in the previous paper [67], and we confirmed that it is also connected to TS2 in the present study. In Figure 4b, interactions a–j are shown for IC1*; in this case, the interactions g and h are O–H covalent bonds as a result of the double proton transfer. In the previous study, the NH_2_ group on the five-membered ring of IC1* was assumed to be readily protonated by the solvent water, and an NH_3_ molecule was released from the protonated intermediate. However, in the mechanism described here, an NH_3_ molecule is released after a proton exchange between the OH group on the five-membered ring and the H_2_PO_4_^−^ ion and subsequent conformational changes without an external proton source. This proton exchange occurs along the two hydrogen bonds between the OH group and H_2_PO_4_^−^ (2.118 and 1.698 Å, see Figure 5). The two transannular hydrogen bonds inside the 15-membered ring of IC1* (2.117 and 1.595 Å, see Figure 5) are also noteworthy. These hydrogen bonds, formed between the Gly C=O group and the NH groups directing to the inside of the 15-membered ring, are maintained over the entire reaction course from RC* to PC. Thus, the overall structure of the reacting system is not largely changed during the entire process.

**Figure 7:**
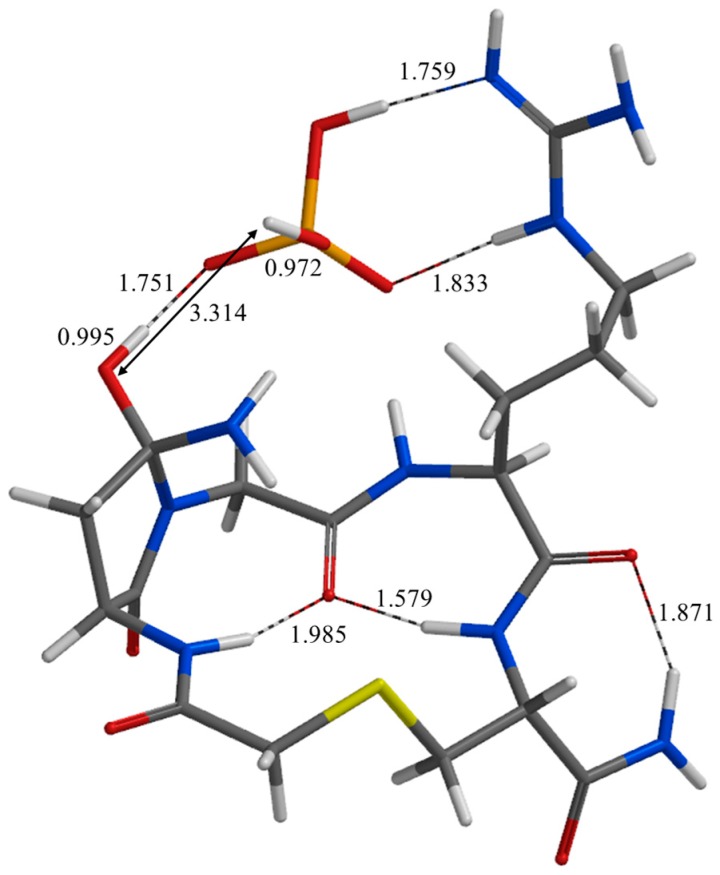
The geometry of IC2 (intermediate complex 2) lying between TS2 (Figure 6) and TS3 (Figure 8). Selected interatomic distances are shown in Å. Grey: carbon; white: hydrogen; blue: nitrogen; red: oxygen; orange: phosphorus; yellow: sulfur.

Figure 6 shows the geometry of TS2. This transition state is for the above-mentioned proton exchange, and connects IC1* and IC2 (intermediate complex 2, Figure 7). The local activation barrier corresponding to TS2 is 33.7 kJ mol^−1^, and the energy of TS2 is substantially lower than that of TS1*. Since the energies of the transition states appearing after IC2 are substantially lower than that of TS2, we can conclude that the rate-determining step of the entire reaction shown in Figure 3 is the first step (i.e., cyclization to form the tetrahedral intermediate). The barrier associated with TS2 (33.7 kJ mol^−1^) may be compared with the barrier to deammoniation (20.2 kJ mol^−1^) of the previous mechanism [67] (the latter value was obtained after the same corrections as in the present paper). In the proton exchange via TS2, the proton abstraction by the H_2_PO_4_^−^ ion precedes the other proton transfer as may be seen from the relevant interatomic distances shown in Figure 6. Figure 7 shows the geometry of IC2. The energy of IC2 is lower than that of IC1* by 14.7 kJ mol^−1^. It should be noted that, in IC2, the proton transferred to the phosphate moiety is close to the NH_2_ group on the five-membered ring (3.056 Å). This proton is finally transferred to the NH_2_ group on the five-membered ring, resulting in the release of an NH_3_ molecule. However, conformational changes are required before the NH_3_ release.

**Figure 8:**
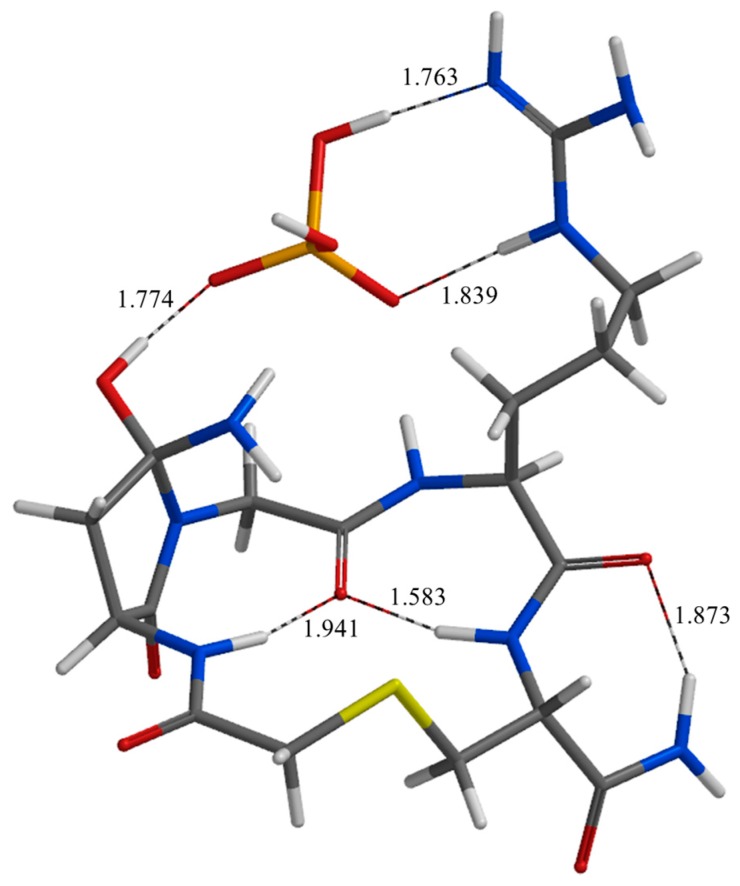
The geometry of TS3 (transition state 3) connecting IC2 (Figure 7) and IC3 (Figure 9). Selected interatomic distances are shown in Å. Grey: carbon; white: hydrogen; blue: nitrogen; red: oxygen; orange: phosphorus; yellow: sulfur.

**Figure 9:**
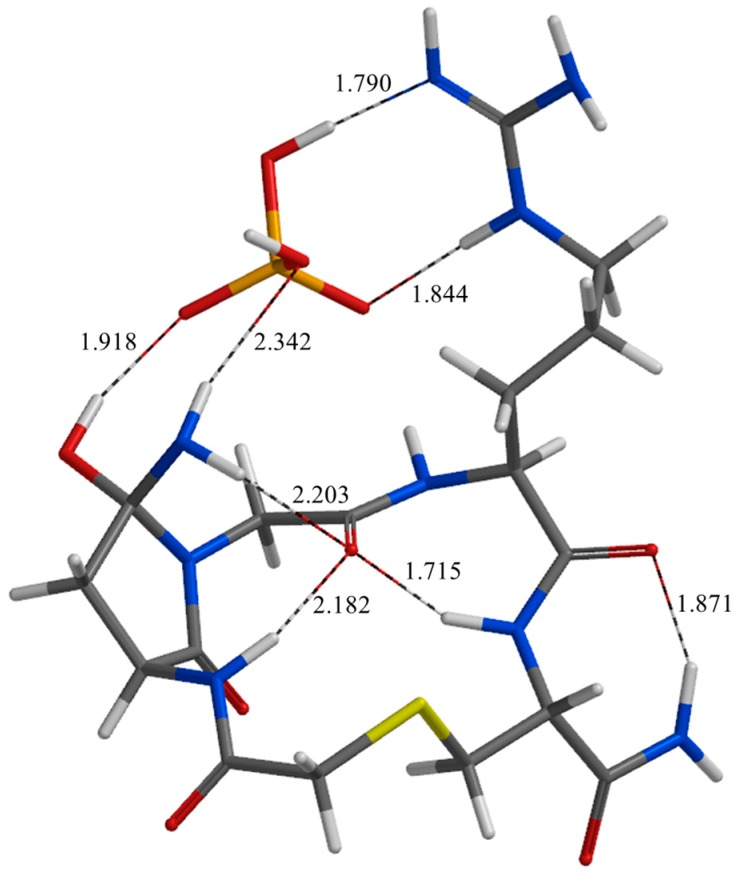
The geometry of IC3 (intermediate complex 3) lying between TS3 (Figure 8) and TS4 (Figure 10). Selected interatomic distances are shown in Å. Grey: carbon; white: hydrogen; blue: nitrogen; red: oxygen; orange: phosphorus; yellow: sulfur.

Figure 8 shows the geometry of TS3 (transition state 3) which connects IC2 and IC3 (intermediate complex 3, Figure 9). This transition state is for a conformational change of the 15-membered ring. In the gas phase, IC2 and TS3 are located on an extremely flat region of the potential energy surface, i.e., their total energies (electronic plus nuclear repulsion energies, both 6-31G(d) and 6-31+G(d,p)) are almost identical. As a result, the relative energy of TS3 became lower than that of IC2 after the thermal and SM8 corrections as shown in Figure 3. The relative energies of IC3 with respect to IC2 and TS3 are −7.3 and −3.8 kJ mol^−1^, respectively. Therefore, we may say that the conversion of IC2 to IC3 is almost barrierless. The conformational change of the 15-membered ring through TS3 is not drastic (and this is the reason for the above-mentioned flatness of the potential energy surface), although it is the largest in the entire course from RC* to PC and is required for the last deammoniation step to occur. In going from IC2 to IC3, the dihedral angles *θ*_1_, *θ*_5_, *θ*_7_, *θ*_13_, and *θ*_14_ change by 38°, 41°, 35°, 38°, and 54°, respectively, as may be seen from Table 2; the changes in the other dihedral angles are much smaller.

**Figure 10:**
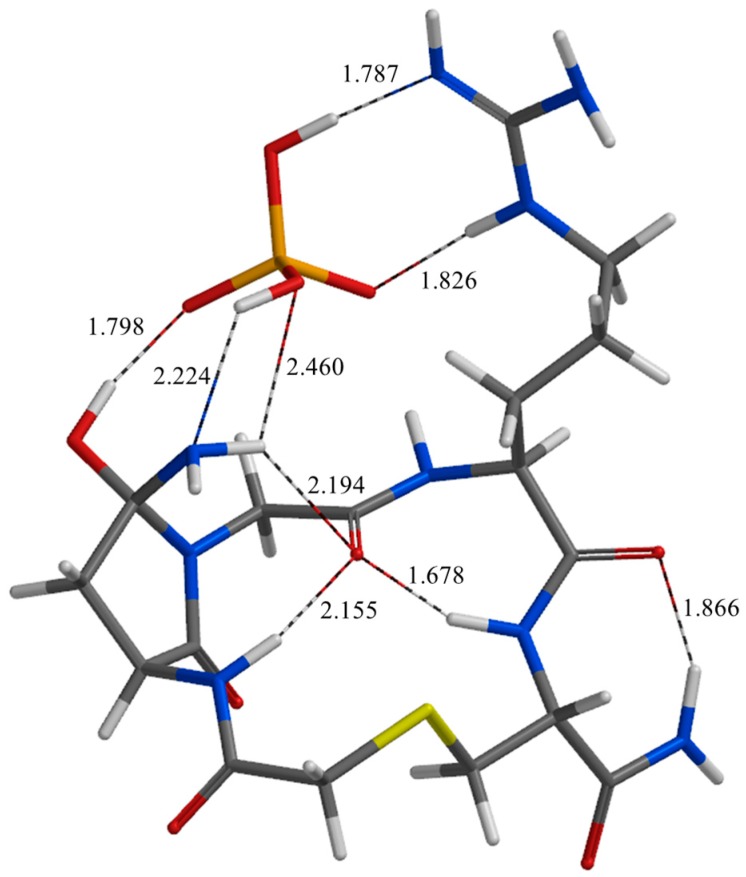
The geometry of TS4 (transition state 4) connecting IC3 (Figure 9) and IC4 (Figure 11). Selected interatomic distances are shown in Å. Grey: carbon; white: hydrogen; blue: nitrogen; red: oxygen; orange: phosphorus; yellow: sulfur.

**Figure 11:**
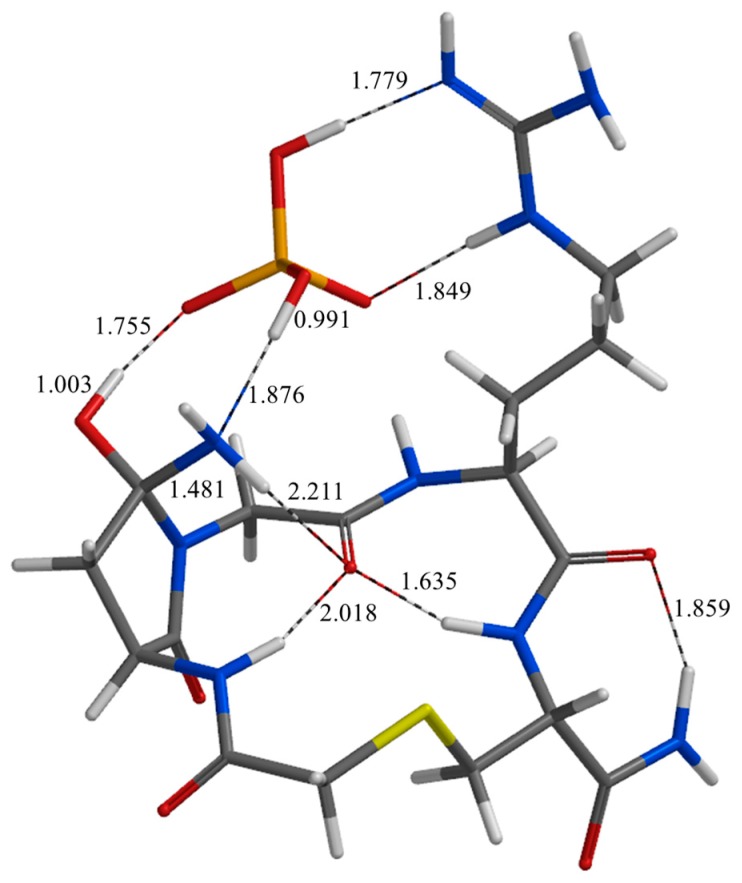
The geometry of IC4 (intermediate complex 4) lying between TS4 (Figure 10) and TS5 (Figure 12). Selected interatomic distances are shown in Å. Grey: carbon; white: hydrogen; blue: nitrogen; red: oxygen; orange: phosphorus; yellow: sulfur.

Figure 10 shows the geometry of TS4 (transition state 4) which connects IC3 and IC4 (intermediate complex 4, Figure 11). The local activation barrier corresponding to TS4 is as low as 13.6 kJ mol^−1^. TS4 is for a concerted rotation of the NH_2_ group on the five-membered ring and the OH group of H_2_PO_4_^−^ hydrogen bonded to it, and this is accompanied by a hydrogen bond reorganization. Most important is the formation of a hydrogen bond between the nitrogen atom of the NH_2_ group and H_2_PO_4_^−^ (1.876 Å, see Figure 11). In IC3, the lone pair of this nitrogen was not involved in a hydrogen bond. In the next and final step, a proton transfer occurs along this newly formed hydrogen bond, leading to the release of an NH_3_ molecule (see below). Simultaneously with the above-mentioned formation of a new hydrogen bond, the hydrogen bond corresponding to interaction f is broken (see Figure 4 and Table 1). The hydrogen bond involving the OH group on the five-membered ring, which were formed in IC2 (1.751 Å, Figure 7) via TS2, is preserved in IC3 (1.918 Å, Figure 9) and IC4 (1.755 Å, Figure 11). This hydrogen bond also plays an important role in the next deammoniation step, i.e., a proton transfer occurs along this hydrogen bond. The energy of IC4 is lower than IC3 and RC* by 21.5 and 14.0 kJ mol^−1^, respectively. The length of the C–N bond which is broken in the next deammoniation step is 1.481 Å in IC4.

**Figure 12:**
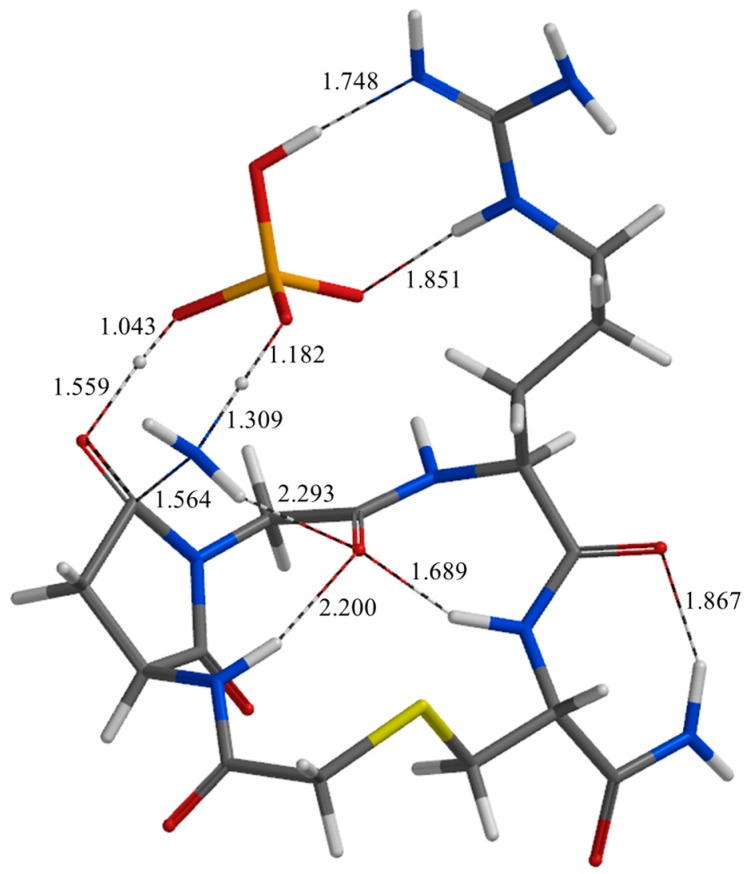
The geometry of TS5 (transition state 5) connecting IC4 (Figure 11) and PC (Figure 13). Selected interatomic distances are shown in Å. Grey: carbon; white: hydrogen; blue: nitrogen; red: oxygen; orange: phosphorus; yellow: sulfur.

**Figure 13:**
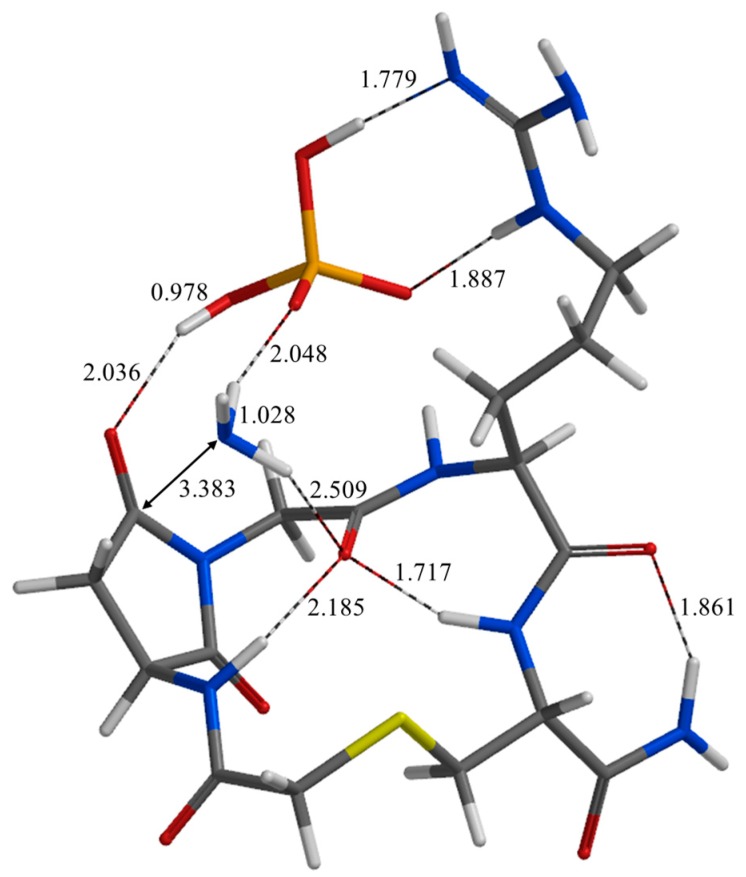
The geometry of PC (product complex). Selected interatomic distances are shown in Å. Grey: carbon; white: hydrogen; blue: nitrogen; red: oxygen; orange: phosphorus; yellow: sulfur.

Figure 12 shows the geometry of TS5 (transition state 5) which connects IC4 and PC (Figure 13). Through TS5, the C–N bond cleavage and a double proton transfer along the two preformed hydrogen bonds between the *gem*-hydroxylamine moiety (HO–C–NH_2_) and H_2_PO_4_^−^ occur, resulting in the release of an NH_3_ molecule and formation of the succinimide moiety. Thus, PC is composed of the succinimide product, an NH_3_ molecule, and an H_2_PO_4_^−^ ion. As may be seen from the relevant interatomic distances shown in Figure 12, the proton abstraction by the H_2_PO_4_^−^ ion precedes that by the NH_2_ group and the C–N bond breaking, although these changes are concerted. The length of the breaking C–N bond is 1.564 Å at TS5 (Figure 12), and the corresponding distance in PC is 3.383 Å (Figure 13), showing that the C–N bond is completely broken. The local activation barrier corresponding to TS5 is 37.5 kJ mol^−1^, and the relative energy of PC with respect to RC* is −29.3 kJ mol^−1^. The entire reaction is cascade-like in that the energies of IC1*, IC2, IC3, IC4, and PC decrease in this order (Figure 3).

Double proton transfers mediated by an H_2_PO_4_^−^ ion were observed in the first, second, and fifth steps through TS1* (Figure 2), TS2 (Figure 6), and TS5 (Figure 12), respectively. All of these are concerted but asynchronous, so that the proton abstraction by an “anionic” oxygen of H_2_PO_4_^−^ precedes the other. Similar results were also obtained in our recent studies on other reactions [52,66,71]. Therefore, it seems to be general that an H_2_PO_4_^−^ ion exert its catalytic ability as both a general base and a general acid by first acting as a general base in the initial stage. It should also be noted that, inside the 15-membered ring, the two transannular hydrogen bonds involving the Gly C=O group were maintained throughout the entire process from RC* to PC. This is in contrast to the previously reported mechanism [67]. These intramolecular hydrogen bonds may contribute to maintain the suitable 15-membered ring structure for the succinimide formation.

## 3. Computational Methods

All calculations were performed by the B3LYP DFT method using Spartan’14 [72]. Geometry optimizations were performed in the gas phase using the 6-31G(d) basis set. The optimized geometries were subjected to vibrational frequency calculations in order to confirm them as either an energy minimum (with no imaginary frequency) or a transition state (with a single imaginary frequency), and correct relative energies for the ZPE. From TS4 and TS5, IRC calculations were successfully performed to confirm the energy minima connected by each of them. However, IRC calculations from TS2 and TS3 failed in the initial stages. For these transition states, full geometry optimizations were performed starting from geometries which were obtained from the transition state by slightly displacing the atoms in the forward and backward directions along the transition vector (i.e., the vibration corresponding to the imaginary frequency). Thermal corrections that give the Gibbs energy at 298.15 K and 1 atm were also performed as implemented in Spartan’14. Furthermore, single-point calculations using the 6-31+G(d,p) basis set were performed to obtain more reliable electronic energies. The hydration Gibbs energies were estimated by the SM8 model [69,70] as implemented in Spartan’14 with the 6-31G(d) basis set. The above procedures are the same as in the previous study [67], except for the thermal corrections and the alternative to the unsuccessful IRC calculations. In the previous paper, the relative energies were ZPE-corrected, but corrections to 298.15 K were not included.

## 4. Conclusions

For the phosphate-catalyzed succinimide formation from c[CH_2_CO–NGRC]–NH_2_, an NGR-motif-containing cyclic peptide, a new mechanism, has been elucidated by the B3LYP DFT method. This comprises five steps. The first step, which was already reported in the previous paper [67], is the cyclization to form a *gem*-hydroxylamine (HO–C–NH_2_) tetrahedral intermediate. In contrast to the previous study, the new mechanism does not require a protonation of the NH_2_ group by the surrounding water to release an NH_3_ molecule, but proceeds from the reactant complex to the product complex without changing stoichiometry. Instead, the proton transfer to the NH_2_ group required for the NH_3_ release occurs internally after three additional steps which includes a proton exchange and conformational changes. Thus, the succinimide formation proceeds in the total of five steps, the first cyclization step being rate-determining. The catalytic phosphate “recognized” by the NGR sequence in the form of H_2_PO_4_^−^ continue to be “trapped” throughout the reaction course, and is involved in a double proton transfer in the first, second, and fifth steps. Since the energies of the transition states of the second to fifth steps are substantially lower than that of the first-step transition state, we can conclude that the four-step decomposition of the initially formed tetrahedral intermediate to give the succinimide product occurs readily by the present mechanism. However, this does not exclude the possibility of the previously proposed mechanism, where the initially formed tetrahedral intermediate is protonated by the surrounding water before the NH_3_ release [67]. Rather, the succinimide formation may occur by more than a single mechanism. We expect that the present results are helpful for a better and deeper understanding of the deamidation behavior of various cyclic NGR-containing peptides. From a manufactural point of view, it will be important to consider the possible specific interactions between the NGR sequence and the buffer species in order to suppress undesired deamidation. From a clinical point of view, it might be important to design NGR-containing peptides considering the interaction with inorganic phosphate to achieve a desired deamidation rate. Finally, the present study and other recent studies of ours [52,66,71] strongly suggest the possibility that inorganic phosphate acts as catalysts in spontaneous, non-enzymatic reactions of amino acid residues in vivo.

## Figures and Tables

**Table 1:** Interatomic distances (Å) in the optimized geometries corresponding to the interactions a–j shown in Figure 4. The geometries labeled by an asterisk (*) are those previously reported [67].

Geometry	a	b	c	d	e	f	g	h	i	j
RC*	1.824	1.789	1.713	2.361	2.421	2.196	1.893	2.235	2.306	2.911
TS1*	1.745	1.828	1.805	2.482	2.584	2.135	1.070	0.978	2.531	2.641
IC1*	1.747	1.840	1.750	2.509	2.738	2.104	1.001	0.977	2.355	2.663
TS2	1.722	1.814	1.794	2.456	2.752	2.472	1.620	1.105	2.359	2.586
IC2	1.759	1.833	1.759	2.508	2.423	2.151	3.314	1.751	2.274	2.822
TS3	1.763	1.839	1.778	2.543	2.418	2.163	3.528	1.774	2.236	2.804
IC3	1.790	1.844	1.932	2.751	2.475	2.342	4.321	1.918	2.161	2.726
TS4	1.787	1.826	1.904	2.688	2.468	2.460	3.345	1.798	2.096	2.811
IC4	1.779	1.849	1.801	2.714	2.435	3.360	3.139	1.755	2.064	2.759
TS5	1.748	1.851	2.030	2.915	2.444	2.968	2.700	1.043	2.232	2.737
PC	1.779	1.887	1.762	2.812	2.297	3.236	3.041	0.978	2.185	3.036

**Table 2:** The heavy-atom dihedral angles *θ*_1_–*θ*_15_ (°) defined for the 15-membered ring as in Figure 1. The geometries labeled by an asterisk (*) are those previously reported [67].

Geometry	*θ*_1_	*θ*_2_	*θ*_3_	*θ*_4_	*θ*_5_	*θ*_6_	*θ*_7_	*θ*_8_	*θ*_9_	*θ*_10_	*θ*_11_	*θ*_12_	*θ*_13_	*θ*_14_	*θ*_15_
RC*	−146	−91	139	−78	−121	165	40	−33	−157	155	−63	159	−131	44	167
TS1*	−165	−89	145	−91	−120	168	20	−18	−165	160	−64	158	−132	60	174
IC1*	−172	−91	149	−85	−133	169	15	−14	−165	159	−63	165	−127	53	−179
TS2	−173	−91	151	−89	−126	170	6	−9	−167	159	−64	165	−130	58	−178
IC2	−152	−92	148	−78	−142	167	31	−23	−163	160	−65	168	−116	31	175
TS3	−142	−95	148	−76	−151	165	43	−25	−162	159	−68	169	−111	20	173
IC3	−114	−105	153	−68	177	174	66	−28	−169	161	−75	175	−78	−23	168
TS4	−115	−100	155	−70	−179	174	66	−32	−166	161	−74	175	−80	−23	166
IC4	−121	−99	151	−74	−171	172	60	−29	−165	161	−73	173	−91	−8	168
TS5	−116	−101	157	−74	180	178	64	−28	−167	161	−76	176	−81	−19	164
PC	−107	−113	147	−63	−180	180	65	−34	−169	162	−73	173	−75	−29	170

## Supplementary Materials

The following are available online, Table S1: Total energies (au), zero-point energies (kJ mol^−1^), and SM8 hydration Gibbs energies (kJ mol^−1^) of the B3LYP/6-31G(d) optimized geometries; Table S2: Cartesian coordinates (Å) of TS2; Table S3: Cartesian coordinates (Å) of IC2; Table S4: Cartesian coordinates (Å) of TS3; Table S5: Cartesian coordinates (Å) of IC3; Table S6: Cartesian coordinates (Å) of TS4; Table S7: Cartesian coordinates (Å) of IC4; Table S8: Cartesian coordinates (Å) of TS5; Table S9: Cartesian coordinates (Å) of PC.
