# Supplementary material for: Phosphate-Catalyzed Succinimide Formation from an NGR-Containing Cyclic Peptide: A Novel Mechanism for Deammoniation of the Tetrahedral Intermediate
Source: Molecules. 2018 Aug 31;23(9):2217. doi: 10.3390/molecules23092217 (PMC6225186; doi:10.3390/molecules23092217)
Supplement: Supplementary file 1 [file molecules-23-02217-s001.pdf]

Supplementary Materials: Phosphate-Catalyzed Succinimide Formation from an NGR-Containing Cyclic Peptide: A Novel Mechanism for Deammoniation of the Tetrahedral Intermediate

Ryota Kirikoshi, Noriyoshi Manabe and Ohgi Takahashi

Table S1. Total energies (au), zero-point energies (kJ mol^{-1}), and SM8 hydration Gibbs energies (kJ mol^{-1}) of the B3LYP/6-31G(d) optimized geometries. The geometries labeled by an asterisk (*) are those previously reported [67].

Geometry	Total Energy (6-31G(d))	Total Energy (6-31+G(d,p))	Zero-Point Energy (6-31G(d))	SM8 Hydration Gibbs energy (6-31G(d))
RC*	−2651.322670	−2651.4960626	1446.1426	−285.0577935
TS1*	−2651.286868	−2651.4624276	1439.8544	−276.1713111
IC1*	−2651.313243	−2651.4879063	1448.9169	−281.5687406
TS2	−2651.297282	−2651.4729734	1437.8046	−276.0368686
IC2	−2651.312073	−2651.4908549	1445.5515	−284.7316989
TS3	−2651.311939	−2651.4909852	1444.8514	−286.9100900
IC3	−2651.313812	−2651.4914760	1448.8380	−293.7571881
TS4	−2651.307375	−2651.4841197	1446.2287	−296.7475414
IC4	−2651.318320	−2651.4956679	1447.3317	−303.2480665
TS5	−2651.297546	−2651.4750425	1431.4004	−303.6178253
PC	−2651.326292	−2651.4998930	1438.0488	−294.0593793

Table S2. Cartesian coordinates (Å) of TS2 (transition state 2).

Atom	*x*	*y*	*z*
6	1.92743584	3.113767271	1.403780519
8	2.405204685	3.160238388	2.530703573
7	0.691867276	2.652470722	1.049331157
6	0.327805033	2.843147357	-0.438020381
7	0.373632857	1.611471377	-1.219271969
1	-1.868604281	3.38876473	0.408468131
6	0.013514875	1.713699351	1.917702939
15	-3.418731285	1.854681918	0.305972825
8	-4.980719955	2.104200005	0.15165422
8	-2.800313525	3.146615304	0.951186363
8	-3.155969812	0.570076228	1.087722064
8	-2.784742253	1.760491921	-1.171979561
6	0.43622234	0.286364269	1.51612844
8	1.658354741	0.035790893	1.466847335
7	-0.573945706	-0.557358783	1.235389324
6	-0.702430713	-1.917443259	0.65423797
6	-1.572940735	-1.802661683	-0.646971392
6	-2.596263315	-2.931544543	-0.84947505
6	-3.767209761	-2.908883244	0.155894692
6	0.504463583	-2.817705457	0.308634589
8	0.232247386	-3.975309546	-0.064829516
7	1.748879155	-2.329877281	0.35470619
6	2.922566285	-2.997414329	-0.206293275
7	-4.566832763	-1.694677522	0.161634117
6	-5.59068505	-1.404123761	-0.683959214
7	-6.153653462	-0.23038049	-0.644677295
7	-6.040917416	-2.452534552	-1.502122299

8	-0.899080211	3.414710345	-0.570911234
1	-2.004861295	2.425416978	-1.220642904
6	1.513321337	3.757937977	-0.867098085
6	2.663315203	3.435561259	0.093152068
7	3.362091133	2.17598401	-0.222949355
6	4.248931592	2.028599609	-1.236292077
8	4.635164765	2.944520613	-1.955964253
6	4.815523382	0.602798138	-1.410441211
16	3.539030386	-0.707830318	-1.696641649
6	4.033216812	-1.967516446	-0.441320223
6	3.463720994	-4.119138801	0.728150374
8	4.564567647	-4.056319653	1.26221502
7	2.613235438	-5.170941016	0.85579535
1	-6.884038417	-0.13631329	-1.347453808
1	-6.627970304	-2.128331819	-2.260429698
1	-5.312473114	-3.06475992	-1.8503101
1	-4.154941349	-0.870584275	0.622014974
1	-4.42172872	-3.772476009	-0.014332151
1	-2.091125125	-3.901411902	-0.789590909
1	-3.378921682	-3.033965592	1.173575667
1	-2.998369857	-2.838989582	-1.869354549
1	-5.463498363	1.236199562	-0.062543781
1	-2.103998942	-0.847793287	-0.619194003
1	-0.902311312	-1.756886686	-1.512864249
1	-1.26246009	-2.500745439	1.39503128
1	-1.509469525	-0.115293673	1.283904446
1	-1.06624598	1.858850653	1.898923009
1	-0.430314368	1.023450853	-1.022474665
1	1.227278538	1.075437629	-1.087469248
1	0.381117345	1.892818498	2.933133625
1	1.197467118	4.796266341	-0.724029128
1	1.766776222	3.603901524	-1.916889676
1	3.397244057	4.23648045	0.204515533
1	5.499961797	0.638119647	-2.25881493
1	5.379734079	0.321211948	-0.51491404
1	3.083178967	1.358670329	0.313867001
1	4.934862641	-2.49043302	-0.765229106
1	4.261655064	-1.466352624	0.503574595
1	2.622134568	-3.470763778	-1.150408629
1	2.841127211	-5.863572542	1.556009112
1	1.650975996	-5.068323308	0.526763858
1	1.873052688	-1.385409573	0.768975621

Table S3. Cartesian coordinates (Å) of IC2 (intermediate complex 2).

Atom	*x*	*y*	*z*
6	2.196172081	2.936853846	1.35012429
8	2.752567777	2.77235041	2.424805054
7	0.860682653	2.767295564	1.082685755
6	0.43331734	3.208905867	-0.296513524
7	0.030469252	2.133045461	-1.184604572
1	-1.389031274	3.758870282	0.209880176
6	0.089100171	1.828142187	1.871450171
15	-3.506098403	1.796139167	0.508724655
8	-5.088186026	2.033375874	0.389047936
8	-2.815094535	3.06252395	0.950448994
8	-3.216883655	0.483133516	1.237299741
8	-3.024897759	1.48512823	-1.064092013
6	0.397684264	0.40531112	1.357440332

8	1.580320934	0.168961779	1.030900436
7	-0.614174765	-0.472411111	1.320860075
6	-0.773812834	-1.809406296	0.687973144
6	-1.498998775	-1.633840481	-0.688268926
6	-2.461913815	-2.769436273	-1.072941031
6	-3.686580132	-2.900404749	-0.141301839
6	0.389882534	-2.80503512	0.474812052
8	0.067062344	-3.989951342	0.262816832
7	1.650043721	-2.355645289	0.430659355
6	2.803340256	-3.079945863	-0.101102027
7	-4.514448474	-1.712145309	-0.028390977
6	-5.566943056	-1.387628967	-0.823083437
7	-6.209905868	-0.271594951	-0.634052424
7	-5.953908929	-2.35557297	-1.767935655
8	-0.580958247	4.154624112	-0.213848761
1	-2.996725802	2.324474513	-1.553315729
6	1.731653547	3.88982981	-0.801017309
6	2.875060162	3.263013753	0.006124277
7	3.31540245	1.962680714	-0.520094794
6	4.372804383	1.804409133	-1.345240698
8	5.062523581	2.722502752	-1.782207421
6	4.758874435	0.351058241	-1.709101838
16	3.42696323	-0.927921964	-1.792605554
6	3.917780712	-2.07618332	-0.429780892
6	3.358651478	-4.139173631	0.895879041
8	4.504125417	-4.095314548	1.329698459
7	2.469551465	-5.120157391	1.193986526
1	-6.942296056	-0.138986657	-1.3291328
1	-6.586216189	-1.979742443	-2.463686157
1	-5.184395342	-2.842188367	-2.213847244
1	-4.145598588	-0.934250734	0.539148895
1	-4.309450995	-3.744507446	-0.459509363
1	-1.931350032	-3.727407905	-1.086342594
1	-3.348297949	-3.155294563	0.86987628
1	-2.810107991	-2.574103004	-2.098368564
1	-5.549122818	1.182909634	0.101392496
1	-2.058347932	-0.697252004	-0.648455942
1	-0.740149682	-1.513522303	-1.471563873
1	-1.446116184	-2.342267567	1.365912818
1	-1.558492309	-0.056627346	1.463354946
1	-0.973498863	2.079989368	1.852953229
1	-0.894983126	1.767997572	-0.964796317
1	0.705171279	1.372703343	-1.207952224
1	0.454040308	1.889253834	2.902324937
1	1.655450132	4.956452023	-0.568627201
1	1.841237502	3.76471617	-1.879288178
1	3.747688446	3.908554905	0.113915235
1	5.244970781	0.404573196	-2.684976953
1	5.512862791	0.009952741	-0.990732781
1	2.83540578	1.139617014	-0.162556546
1	4.814881097	-2.628193356	-0.713521563
1	4.151549835	-1.494407143	0.466684887
1	2.486757642	-3.61745431	-1.005970269
1	2.726014501	-5.770231397	1.924104856
1	1.492333468	-5.014604281	0.911418885
1	1.778826237	-1.350316297	0.649037942

Table S4. Cartesian coordinates (Å) of TS3 (transition state 3).

Atom	*x*	*y*	*z*
6	2.165536493	2.887755612	1.319163372
8	2.7390345	2.638236131	2.368525523
7	0.818751259	2.77744482	1.078263566
6	0.374823516	3.284108853	-0.271895036
7	-0.07402272	2.254678536	-1.193054227
6	0.04586157	1.814838723	1.833532311
15	-3.540823623	1.741608143	0.541605959
8	-5.127674861	1.971377585	0.460481667
8	-2.845097019	3.015475821	0.952523282
8	-3.225757507	0.435311883	1.270001491
8	-3.108198043	1.421850584	-1.044199552
6	0.339160685	0.410641992	1.264375456
8	1.472256212	0.216747777	0.779498668
7	-0.62025403	-0.521646313	1.363266359
6	-0.760530817	-1.836438423	0.680171763
6	-1.458576422	-1.637885513	-0.705806987
6	-2.409071736	-2.772155005	-1.125819775
6	-3.644505076	-2.933624054	-0.213054776
6	0.421113182	-2.81769244	0.482685875
8	0.120147081	-4.006835527	0.265503306
7	1.677145556	-2.349115686	0.467301136
6	2.851697292	-3.039839682	-0.067432314
7	-4.476118053	-1.750971551	-0.072638606
6	-5.551585822	-1.425411383	-0.834704825
7	-6.222298965	-0.33655872	-0.590663918
7	-5.926298131	-2.365848971	-1.812314824
8	-0.615926904	4.247854032	-0.127357723
6	1.6801534	3.95177955	-0.776968383
6	2.828519396	3.272682512	-0.01822177
7	3.261727675	1.999978766	-0.614067309
6	4.41622797	1.837721478	-1.295110283
8	5.195476915	2.741375223	-1.587984106
6	4.790112661	0.392917897	-1.70961406
16	3.483097636	-0.911584885	-1.795047069
6	3.946152649	-2.006425161	-0.377608372
6	3.420408005	-4.100081014	0.920468946
8	4.567243047	-4.050528903	1.35016517
7	2.540806055	-5.090351308	1.214661918
1	5.577271887	0.045799119	-1.03122963
1	5.242774203	0.472898585	-2.700402716
1	4.124073915	-1.392185611	0.510397432
1	4.871482083	-2.536044046	-0.607979694
1	2.555548269	-3.574728712	-0.981262916
1	1.767001227	-1.329506669	0.613937421
1	2.807227788	-5.749139615	1.933123423
1	1.563917943	-5.000601114	0.92587253
1	-1.440624068	-2.390921546	1.331611285
1	-1.567054855	-0.137834662	1.557293033
1	-0.687844536	-1.501256077	-1.474628964
1	-2.024873909	-0.705521382	-0.659239139
1	-1.871723578	-3.726480627	-1.151796023
1	-2.745397374	-2.558812992	-2.151587798
1	-3.317039626	-3.22161072	0.792953423
1	-4.261802765	-3.766957374	-0.567699991
1	-5.148734479	-2.798494562	-2.298635084
1	-6.59022472	-1.984075022	-2.47473353

1	-6.962224036	-0.19244107	-1.27556371
1	-5.58328765	1.118810649	0.171835947
1	-4.118283035	-0.988000231	0.521162343
1	-3.151254644	2.250262275	-1.550477747
1	1.633987996	5.010845919	-0.505128424
1	1.763217395	3.863280748	-1.861309492
1	3.708952069	3.903763489	0.109385792
1	2.741601275	1.171055284	-0.335840871
1	0.576303098	1.474754731	-1.235763242
1	-1.001342042	1.903280472	-0.958375139
1	-1.016335381	2.069913069	1.825368112
1	0.410364046	1.832339201	2.866527443
1	-1.433963888	3.831296992	0.251342306

Table S5. Cartesian coordinates (Å) of IC3 (intermediate complex 3).

Atom	*x*	*y*	*z*
6	-2.222428939	-2.361306247	1.201533151
8	-2.774819959	-1.606437747	1.993918457
7	-0.881561944	-2.597348647	1.119745142
6	-0.454951271	-3.509037868	-0.001740044
7	0.241417093	-2.84832022	-1.090111696
6	0.011716818	-1.618751459	1.684183987
15	3.519317185	-1.749200028	0.559844697
8	5.101549376	-2.050191479	0.512589219
8	2.765739419	-3.010903381	0.908942978
8	3.233838226	-0.453081508	1.312238934
8	3.16406929	-1.370295753	-1.031196736
6	-0.068813553	-0.330714736	0.849771332
8	-0.843568655	-0.249375221	-0.120859864
7	0.670402747	0.711004958	1.282454908
6	0.817415188	1.976204792	0.539021314
6	1.541683282	1.796890271	-0.831957748
6	2.558640254	2.907399135	-1.138477092
6	3.773338259	2.935061197	-0.182875802
6	-0.444805124	2.855907596	0.377309391
8	-0.285364288	4.081195621	0.241888062
7	-1.648599973	2.2485137	0.37746842
6	-2.934862157	2.855673155	0.018372077
7	4.519801134	1.693202667	-0.083166791
6	5.625165496	1.355309921	-0.794398665
7	6.266722243	0.254032724	-0.530542903
7	6.054111012	2.298791382	-1.748357056
8	0.349204041	-4.531934432	0.499729018
6	-1.82669945	-4.06793477	-0.459126103
6	-2.898239625	-3.074969996	0.019929109
7	-3.242112472	-2.007049215	-0.931094285
6	-4.537368547	-1.703695866	-1.211543454
8	-5.493800514	-2.43145444	-0.96294702
6	-4.806944272	-0.392887711	-1.98191795
16	-3.590990467	0.984339067	-1.953572441
6	-3.934751225	1.733778059	-0.30144469
6	-3.515474348	3.751431465	1.152194572
8	-4.634927173	3.576156684	1.618429467
7	-2.692136117	4.761882941	1.526187317
1	5.586597276	-1.212930321	0.23741582
1	7.024223255	0.097295946	-1.193437713
1	6.747457077	1.915747636	-2.379257581
1	5.300858797	2.733702481	-2.270371055

1	4.132485846	0.943069957	0.509441097
1	3.434159236	3.188967572	0.828985169
1	4.455350737	3.74250537	-0.473314529
1	2.914156038	2.768594238	-2.170624071
1	2.066036234	3.887801408	-1.099519975
1	1.452350598	2.585754877	1.186065574
1	0.799387531	1.741386355	-1.635459021
1	2.058283183	0.833715242	-0.816106012
1	1.551384928	0.405886363	1.718842642
1	-1.743283846	4.818958524	1.15241469
1	-2.978756357	5.329788373	2.310823749
1	-2.790298477	3.506555105	-0.857151906
1	-1.612050261	1.232712382	0.272751785
1	-3.878250224	0.958502618	0.470822645
1	-4.943738981	2.148145891	-0.289441399
1	-5.786105797	-0.035314965	-1.652086861
1	-4.906081858	-0.65231404	-3.042245416
1	-2.564277763	-1.251453473	-1.013324531
1	-3.838251296	-3.549173465	0.304030258
1	-1.972317761	-5.031079646	0.039689727
1	-1.831783688	-4.225273314	-1.538957872
1	3.306986925	-2.162273487	-1.574637494
1	-0.230849192	-1.981531499	-1.335851735
1	1.180027954	-2.590263151	-0.786612202
1	-0.314084455	-1.378963949	2.700902269
1	1.033125548	-2.008753456	1.723718611
1	1.245959608	-4.153730615	0.659042113

Table S6. Cartesian coordinates (Å) of TS4 (transition state 4).

Atom	*x*	*y*	*z*
6	-2.208581056	-2.39703964	1.208818375
8	-2.79864737	-1.684418158	2.0101424
7	-0.857534447	-2.587415275	1.14234031
6	-0.402871602	-3.484647467	0.032727267
7	0.192444837	-2.756182137	-1.106909289
6	0.001841353	-1.61702376	1.778590911
15	3.439115473	-1.731745531	0.60238776
8	5.004786146	-2.031835622	0.389452525
8	2.745488825	-2.962081695	1.147905979
8	3.263369434	-0.392953995	1.325708966
8	2.853765466	-1.479141444	-0.933415906
6	-0.062520613	-0.307544835	0.976494014
8	-0.839419069	-0.219687768	0.006470136
7	0.692031674	0.723078577	1.404059982
6	0.827852549	1.989343027	0.651986388
6	1.510372821	1.797953842	-0.7387072
6	2.498653167	2.919273515	-1.093428282
6	3.757646644	2.955139504	-0.196633269
6	-0.43124619	2.878005274	0.502776609
8	-0.270150557	4.107290411	0.417088657
7	-1.632451611	2.268362202	0.431476075
6	-2.901973277	2.864095562	0.003197828
7	4.509545786	1.715554076	-0.127004281
6	5.530987756	1.349609533	-0.944094483
7	6.162844342	0.230760915	-0.743533082
7	5.894365454	2.285578437	-1.933092882
8	0.478557417	-4.43742023	0.516753263
6	-1.743767711	-4.134141281	-0.379600543

6	-2.831729763	-3.117276787	0.002972753
7	-3.093909647	-2.053846274	-0.984174873
6	-4.371707232	-1.740977825	-1.342104163
8	-5.339286332	-2.473323822	-1.164149133
6	-4.597020548	-0.417969493	-2.107171514
16	-3.392756853	0.965811707	-1.994094245
6	-3.865439697	1.728144615	-0.378792477
6	-3.559388988	3.7481907	1.103471959
8	-4.7075472	3.56543775	1.491188909
7	-2.76492268	4.756249859	1.538990677
1	5.476510497	-1.207351418	0.064265645
1	6.846094774	0.051006961	-1.477331251
1	6.51417741	1.8843134	-2.626291353
1	5.108814758	2.742175062	-2.383992397
1	4.147748611	0.967331699	0.487827999
1	3.466516076	3.21056595	0.829435461
1	4.422885775	3.763404029	-0.522940339
1	2.804118194	2.783341582	-2.141974714
1	1.999980703	3.895655799	-1.032494644
1	1.483393847	2.596281998	1.279919248
1	0.742503512	1.720785011	-1.516003231
1	2.040264975	0.841700514	-0.725304139
1	1.598031229	0.40491039	1.788621051
1	-1.792803175	4.816692343	1.229421435
1	-3.10420051	5.318280753	2.306664321
1	-2.716701376	3.520862847	-0.860440863
1	-1.581238718	1.253996899	0.31252343
1	-3.854321358	0.961068942	0.403513486
1	-4.87719433	2.130241043	-0.444857575
1	-5.595177887	-0.069032223	-1.829141454
1	-4.636313835	-0.664024341	-3.174635889
1	-2.418855673	-1.289321544	-0.995706402
1	-3.794009062	-3.572768032	0.237165109
1	-1.872349909	-5.041517062	0.218479893
1	-1.751473587	-4.415413847	-1.436247208
1	2.361725716	-2.277011021	-1.208031811
1	-0.487243815	-2.679567934	-1.860085723
1	0.415347211	-1.798318007	-0.85675242
1	-0.382070554	-1.411535108	2.782774307
1	1.023595519	-2.003919519	1.862625474
1	1.358746838	-3.998352489	0.663426198

Table S7. Cartesian coordinates (Å) of IC4 (intermediate complex 4).

Atom	*x*	*y*	*z*
6	2.187123188	2.572654508	1.252632051
8	2.771839066	2.030230865	2.176772403
7	0.824730887	2.659053672	1.099553611
6	0.382019787	3.408131503	-0.096578605
7	-0.215999575	2.494874574	-1.096917569
1	-1.399698013	3.925027253	0.485574022
6	0.002284353	1.668329112	1.756787395
15	-3.428680376	1.747830445	0.594535962
8	-4.995700707	2.037809549	0.400329593
8	-2.72291626	2.976455172	1.140711102
8	-3.24442586	0.424965932	1.362978979
8	-2.902546798	1.5057794	-0.947371138
6	0.137102208	0.349581908	0.984323876
8	1.045010813	0.234303401	0.132761192

7	-0.696374511	-0.64553219	1.320161552
6	-0.838873245	-1.92471522	0.591216539
6	-1.530227557	-1.728183291	-0.794009487
6	-2.516999143	-2.848780957	-1.155512103
6	-3.746918982	-2.925687036	-0.221859654
6	0.386192346	-2.858677021	0.436481854
8	0.165650047	-4.074831014	0.299189628
7	1.615530651	-2.308396116	0.413444217
6	2.860810097	-2.951522945	-0.015926227
7	-4.512644028	-1.699344279	-0.095089448
6	-5.571423226	-1.337344712	-0.865655296
7	-6.215451933	-0.235513926	-0.619124598
7	-5.956114729	-2.261924791	-1.858073116
8	-0.55658658	4.381522141	0.18935107
1	-1.969748762	1.837431384	-0.986190552
6	1.714348483	4.060157447	-0.561141075
6	2.833279131	3.160891511	-0.012024941
7	3.183119876	2.004564314	-0.852232732
6	4.464626903	1.761215931	-1.23285872
8	5.381008915	2.574026053	-1.160705826
6	4.775075398	0.383755138	-1.863310904
16	3.523189959	-0.966443915	-1.896322583
6	3.898462094	-1.85874197	-0.319866984
6	3.445501091	-3.913414787	1.06058567
8	4.58533786	-3.793718197	1.495677042
7	2.598014066	-4.907356498	1.421616737
1	-4.138289318	-0.955411473	0.518374519
1	-5.48177512	1.204086765	0.126277267
1	-0.418695073	3.055793222	-1.923410954
1	0.423346384	1.745941288	-1.356098362
1	1.747182232	4.180871646	-1.647174301
1	1.782482872	5.04957767	-0.097847856
1	3.760773089	3.693363133	0.200281788
1	2.562409634	1.199332139	-0.783144604
1	5.029973222	0.572535901	-2.911272794
1	5.689992568	0.01854918	-1.3871612
1	3.922493204	-1.142094045	0.507586813
1	4.883609715	-2.321231065	-0.390857158
1	2.885324785	-5.511273984	2.178862853
1	1.630018793	-4.900264266	1.092119595
1	2.662413172	-3.557293641	-0.912992257
1	1.613078566	-1.284038814	0.345478317
1	-1.507565429	-2.507087014	1.228563282
1	-0.765151461	-1.64117923	-1.574372075
1	-2.065542965	-0.774252307	-0.767596525
1	-1.620884792	-0.29758902	1.655233594
1	0.401226089	1.510417942	2.763775614
1	-1.03491448	2.010194214	1.831428364
1	-2.85555296	-2.680389477	-2.189009389
1	-2.008944607	-3.821293108	-1.139245475
1	-3.420415285	-3.208664307	0.785927866
1	-4.41151808	-3.733231276	-0.550978493
1	-5.179376541	-2.691963624	-2.348696765
1	-6.608874678	-1.857581245	-2.51853131
1	-6.929546911	-0.053852297	-1.322405235

Table S8. Cartesian coordinates (Å) of TS5 (transition state 5).

Atom	*x*	*y*	*z*
6	2.16462237	-2.333545621	-1.244897219
8	2.763890109	-1.593159765	-2.019394331
7	0.817766109	-2.503113973	-1.170559202
6	0.326613744	-3.486468981	-0.119893122
7	-0.200341102	-2.653214708	1.094874631
1	-1.955413488	-3.560447062	-0.885216399
6	-0.019026462	-1.513736878	-1.788993539
15	-3.293332899	-1.747144072	-0.494713111
8	-4.768908934	-2.136282789	-0.009568439
8	-2.794050817	-3.056665599	-1.248193565
8	-3.327994542	-0.521196757	-1.406828154
8	-2.386811542	-1.511045165	0.764595675
6	0.004712795	-0.249846576	-0.917354092
8	0.768940462	-0.182950901	0.06142286
7	-0.731053486	0.797419424	-1.343915415
6	-0.851851299	2.064321165	-0.598161817
6	-1.597654849	1.905567972	0.765313274
6	-2.670901559	2.982775838	0.988773422
6	-3.873497083	2.880750084	0.024004036
6	0.429614064	2.914246523	-0.408116327
8	0.290487884	4.141005158	-0.26530358
7	1.62175978	2.28378148	-0.38578814
6	2.909381516	2.847182892	0.034615108
7	-4.585583316	1.616867822	0.048765311
6	-5.575428348	1.278388591	0.915177044
7	-6.115731889	0.096827486	0.863432286
7	-6.015016577	2.296612638	1.781777563
8	-0.627918979	-4.280805384	-0.498496364
1	-1.35067388	-2.069189835	0.870587336
6	1.700884305	-4.118944373	0.296761101
6	2.796201625	-3.108093469	-0.07932843
7	3.103456797	-2.069396221	0.925302413
6	4.388834074	-1.80537546	1.290714086
8	5.331524271	-2.572004786	1.12550278
6	4.657727382	-0.48091034	2.041155818
16	3.456133338	0.910007945	1.981609223
6	3.868042686	1.688333222	0.356839413
6	3.555363375	3.761352444	-1.048482299
8	4.698974024	3.589321326	-1.45411435
7	2.758143071	4.781581481	-1.448408164
1	-6.818423201	-0.029919669	1.589266484
1	-6.561055551	1.932998556	2.552997911
1	-5.282893829	2.908552539	2.123103696
1	-4.216014962	0.84671081	-0.532247606
1	-4.575645822	3.701236876	0.217797026
1	-2.22299678	3.980391077	0.887435437
1	-3.53061912	3.027958944	-1.00654828
1	-3.030045927	2.901196438	2.02579474
1	-5.30210016	-1.320109156	0.242892464
1	-2.052922211	0.910358606	0.789055509
1	-0.872453069	1.933323153	1.585982921
1	-1.457207714	2.695397412	-1.253566853
1	-1.61252102	0.497466087	-1.782232776
1	-1.026390628	-1.894593914	-1.960601547
1	0.461051803	-1.970029232	1.465115065
1	-0.39958198	-3.348061928	1.814728026

1	0.412764397	-1.242475116	-2.75830451
1	1.822750504	-5.023985597	-0.307206255
1	1.720078678	-4.405964157	1.352536279
1	3.746563143	-3.5693002	-0.350807972
1	4.746604716	-0.724980119	3.105887646
1	5.643824975	-0.136422263	1.717312445
1	2.472963371	-1.269223005	0.930547513
1	4.887678921	2.074586936	0.384288618
1	3.811349581	0.933777234	-0.43566655
1	2.75173632	3.47692259	0.923490974
1	3.096229476	5.374257964	-2.193070699
1	1.796553211	4.851103962	-1.110469381
1	1.557989134	1.268233134	-0.292040135

Table S9. Cartesian coordinates (Å) of PC (product complex).

Atom	*x*	*y*	*z*
6	2.361287657	-2.308044739	-1.206732433
8	2.933336162	-1.539431267	-1.950882897
7	1.015742271	-2.66764442	-1.318736918
6	0.631617612	-3.637356747	-0.387887385
7	-0.201922908	-2.059665711	2.485978812
1	-2.264111832	-3.472114472	-1.016318534
6	0.087645908	-1.712703819	-1.894763946
15	-3.391170266	-1.666935599	-0.642875106
8	-4.938558316	-2.064750239	-0.417598716
8	-2.885729524	-2.942399505	-1.554172299
8	-3.325628219	-0.404084115	-1.523900222
8	-2.576166332	-1.641581528	0.632764004
6	0.056039642	-0.48280917	-0.958065457
8	0.854174996	-0.422085336	-0.005091004
7	-0.757400189	0.51682354	-1.331557486
6	-0.926374233	1.770162807	-0.570967251
6	-1.584834753	1.562364095	0.825537939
6	-2.548068125	2.694293341	1.219191679
6	-3.7782464	2.845065467	0.294033678
6	0.296491498	2.705629585	-0.460422959
8	0.095370838	3.931091225	-0.400344122
7	1.523644522	2.147439523	-0.402194988
6	2.775144633	2.840021825	-0.081607183
7	-4.579126646	1.64819535	0.110225053
6	-5.58515711	1.237848418	0.928075975
7	-6.225070044	0.134433409	0.6792962
7	-5.928274575	2.119125935	1.973360227
8	-0.468790047	-4.150457552	-0.336131468
1	-1.024024282	-2.13682556	1.873767713
6	1.814450342	-3.918386313	0.528339481
6	2.940002076	-2.98050629	0.053213252
7	3.260167258	-1.909004859	0.988475897
6	4.542558957	-1.533171262	1.235183604
8	5.529916286	-2.188295413	0.915902818
6	4.740266674	-0.243283452	2.056650797
16	3.475974499	1.086815834	1.982200507
6	3.827806173	1.795922166	0.315230263
6	3.321761398	3.677228384	-1.276111539
8	4.437334188	3.492521426	-1.748643957
7	2.473724126	4.648441388	-1.696550286
1	-4.229790452	0.94115232	-0.55723752
1	-5.45516351	-1.268927757	-0.096451284

1	-0.804190728	1.469831054	1.589391882
1	-0.570603926	-2.03426638	3.437384977
1	0.148042705	-1.119851517	2.299457848
1	1.466449092	-3.667814269	1.538674654
1	2.087191175	-4.977793489	0.494283477
1	3.87866634	-3.488830819	-0.174583387
1	2.514491188	-1.238549732	1.157515461
1	4.787629509	-0.524650163	3.115041552
1	5.722249243	0.151233816	1.78357767
1	3.842620885	0.990501131	-0.427093221
1	4.811572802	2.267268936	0.31840645
1	2.592388451	3.542707743	0.744996138
1	2.728427239	5.155402534	-2.532590831
1	1.519911855	4.684549219	-1.331054297
1	1.531503048	1.133853551	-0.265838108
1	0.464109049	-1.391520734	-2.869247801
1	-0.899993871	-2.160951531	-2.022418981
1	-1.676872742	0.188232328	-1.713681298
1	-2.111144423	0.601655798	0.804867693
1	-1.614293344	2.347627015	-1.19127524
1	-2.887939742	2.500466609	2.248062574
1	-2.019205426	3.656322005	1.236921473
1	-3.449425746	3.165849473	-0.700481168
1	-4.419230895	3.6543789	0.66476646
1	-5.135069448	2.561770226	2.424190631
1	-6.519483776	1.672126667	2.663542131
1	-6.909565121	-0.07272869	1.404657623
